# The Paradox of Long-Term Pharmacotherapy in Psychiatry: Tardive Syndromes

**DOI:** 10.1192/j.eurpsy.2026.11778

**Published:** 2026-07-31

**Authors:** I. Hacisalihoglu Aydin, R. S. El-Mallakh

**Affiliations:** Department of Psychiatry and Behavioral Sciences, Mood Disorders Research Program, Depression Center, University of Louisville School of Medicine, Louisville, United States

## Abstract

**Introduction:**

Long-term use of some psychotropics can paradoxically lead to persistent adverse effects which oppose their initial therapeutic goals, collectively termed tardive syndromes.

**Objectives:**

To examine the clinical and preclinical evidence behind the tardive syndrome or oppositional tolerance concept.

**Methods:**

A scoping review of the data regarding oppositional tolerance using benzodiazepines (BNZ) as examples of agonists and antipsychotics (AP) as examples of antagonists.

**Results:**

Prolonged use of BNZs and APs can cause oppositional tolerance that presents as worsening of anxiety or psychosis, respectively. This phenomenon is related to the up or down regulation of receptors, neurotransmitter (NT) release, or synaptic number between neurons (Image 1). Adaptive alterations are less likely to happen with partial agonist APs, which provides a clue as to how clinicians may mitigate the process. This must be recognized among potential complications of long-term use of psychotropic medications.

**Image 1:**
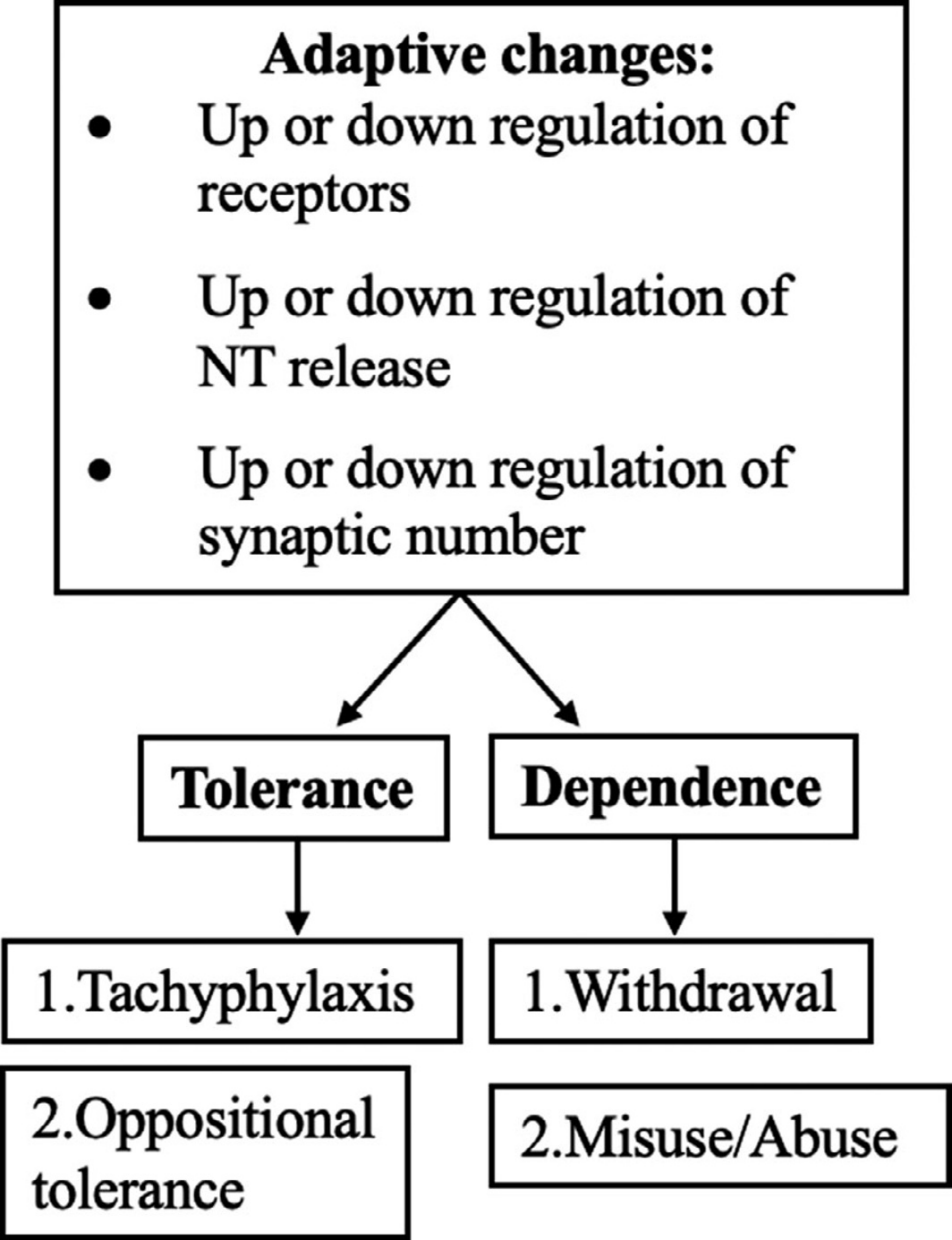

**Conclusions:**

Adaptive responses of neurons and circuits to pharmacological stimuli can result in a paradoxical oppositional response which complicates long term treatment of chronic conditions. Understanding the long-term effects of BNZs and APs is essential for clarifying why certain patients are more susceptible to receptor supersensitivity or resistance. This can ultimately enable safer and more effective use of these treatments.

**Disclosure of Interest:**

I. Hacisalihoglu Aydin: None Declared, R. S. El-Mallakh Speakers bureau of: Axsome, Intracellular, Lundbeck, Otuka, and Vanda.

